# Spatial Interpolation of Gravimetric Soil Moisture Using EM38-mk Induction and Ensemble Machine Learning (Case Study from Dry Steppe Zone in Volgograd Region)

**DOI:** 10.3390/s22166153

**Published:** 2022-08-17

**Authors:** Anatoly Zeyliger, Andrey Chinilin, Olga Ermolaeva

**Affiliations:** 1 Saratov State University of Genetics, Biotechnology and Engineering Named after N.I. Vavilov, 410012 Saratov, Russia; 2 Department of Soil Geography, FRC “V.V. Dokuchaev Soil Science Institute”, 119017 Moscow, Russia; 3 Department of Applied Informatics, Russian State Agrarian University—Moscow Timiryazev Agricultural Academy, 127550 Moscow, Russia

**Keywords:** soil sensor, proximal soil sensing, precision irrigated agriculture, soil moisture mapping, spatial interpolation, apparent electrical conductivity, soil surface, hydrological parameters, water runoff, microrills

## Abstract

The implementation of the sustainable management of the interaction between agriculture and the environment requires an increasingly deep understanding and numerical description of the soil genesis and properties of soils. One of the areas of application of relevant knowledge is digital irrigated agriculture. During the development of such technologies, the traditional methods of soil research can be quite expensive and time consuming. Proximal soil sensing in combination with predictive soil mapping can significantly reduce the complexity of the work. In this study, we used topographic variables and data from the Electromagnetic Induction Meter (EM38-mk) in combination with soil surface hydrological variables to produce cartographic models of the gravimetric soil moisture for a number of depth intervals. For this purpose, in dry steppe zone conditions, a test site was organized. It was located at the border of the parcel containing the irrigated soybean crop, where 50 soil samples were taken at different points alongside electrical conductivity data (EC_a_) measured in situ in the field. The modeling of the gravimetric soil moisture was carried out with the stepwise inclusion of independent variables, using methods of ensemble machine learning and spatial cross-validation. The obtained cartographic models showed satisfactory results with the best performance R^2^_cv_ 0.59–0.64. The best combination of predictors that provided the best results of the model characteristics for predicting gravimetric soil moisture were geographical variables (buffer zone distances) in combination with the initial variables converted into the principal components. The cartographic models of the gravimetric soil moisture variability obtained this way can be used to solve the problems of managed irrigated agriculture, applying fertilizers at variable rates, thereby optimizing the use of resources by crop producers, which can ultimately contribute to the sustainable management of natural resources.

## 1. Introduction

Currently, in scientific research and in practice, there is a high demand for soil moisture spatial data for solving a wide range of problems, including the prediction of climate change, forecasting droughts and floods, carbon cycle modeling, as well as maintaining and developing precision farming. This was reflected by the UN World Meteorological Organization as the most important climate variable in the Global Climate Observing System [1]. Despite this, continuous spatial data of the soil moisture, obtained from satellite radar monitoring of the earth’s surface, are available today only for the surface layers (0–5 cm) of soil. However, the low spatial resolution of these data and their limited cover of soil depth does not meet the needs of many problems at regional and especially local levels.

The Volgograd oblast, where the field experiment was conducted, is an important region for irrigated agriculture in Russia. Irrigation in this region began at the end of the 19th century and reached its peak in the second half of the 20th century. In 1989, 345,200 hectares were irrigated, which makes up about 5% of the agricultural area. The Russian economic crisis in the 1990s–2000s caused a reduction in the area of irrigated land to 259,400 ha (3.2% of the agricultural land area), and at the beginning of 2018 the irrigated area was 180,700 ha (2.2% of agricultural land) [2,3].

One of the reasons for the protracted restoration of irrigated agriculture are the lack of adequate data necessary for its management. Spatial information about soil moisture obtained using predictive models is important to support the intensification and digitalization of irrigated agriculture in the semi-arid conditions of such regions. However, such predictive models are absent even in the global soil initiatives, such as SoilGrids [4] or GlobalSoilMap [5], which makes it difficult to provide adequate support for the intensification of crop farms or farmers.

Obtaining such predictive models is possible by combining ground-based proximal soil sensing (PSS) techniques, remote sensing technologies and machine learning algorithms, which allow for the implementation of appropriate strategies for managed irrigated agriculture [6]. The transition to such strategies is impossible without the objective control of water irrigation efficiency, the implementation of irrigation by robotic sprinklers, management and the control of irrigation water consumption, as well as control over its quality.

Recent developments in automated predictive soil mapping and advances in remote sensing data are enabling soil scientists to rethink soil mapping methods based on “factorial” modeling [7]. In this regard, McBratney et al. [8], developing the theory of V.V. Dokuchaev, proposed a new soil mapping paradigm based on quantitative modeling. This paradigm is based on the scorpan model, which includes spatial data on soil formation factors. Spatial data characterizing soil formation factors can involve: satellite information of different spectral, radiometric and spatial resolutions; climate models; digital elevation and terrain models; land use maps; vegetation maps; soil spectroscopy data; and PSS data [9]. PSS is one of the digital soil mapping methods that is an alternative to the laboratory chemical and physical methods of soil analysis and uses various sensors to measure and determine soil properties [10]. Examples of such an alternative can be non-invasive, mobile, active or passive geophysical instruments, such as gamma-ray spectrometers, electromagnetic inductometers or ground penetrating radars. These tools provide extensive, direct or indirect data on the physical and chemical properties of soils in situ, which can then be used as input variables for predictive soil mapping. Electromagnetic inductometers measure electrical conductivity (EC_a_), the values of which can be used to produce cartographic models of such soil properties as soil texture [11,12,13], cation exchange capacity [14], soil moisture [15,16,17] and salinity [18,19,20].

In recent decades, machine learning approaches have become central to predictive soil mapping research, as they offer powerful tools to overcome vulnerability in traditional mapping approaches [21,22]. One of these limitations was the inability of conventional cartographic models to represent nonlinear relationships, which is easily implemented in modern machine learning algorithms [23,24]. Machine learning has been considered suboptimal for a long time regarding spatial interpolation problems as compared to classical geostatistical methods, such as kriging, since it basically ignores spatial dependence structure in the data. The incorporation of the spatial dependence structure in machine learning became possible due to the so-called “geographical variables”: buffer distances, distances within watershed and oblique coordinates as independent variables [25,26,27,28]. This made it possible to increase the efficiency of prediction performance and produce maps that visually appear as though they have been produced by kriging.

Given the above, the purpose of this study is to obtain information on the spatial and vertical distribution of the gravimetric soil moisture of the test area. To achieve this goal, a number of tasks were solved: (1) evaluating the performance of ensemble machine learning and spatial cross-validation, (2) quantifying the prediction error and (3) evaluating the variability of soil moisture with depth.

## 2. Materials and Methods

### 2.1. Study Area

**Figure 1:**
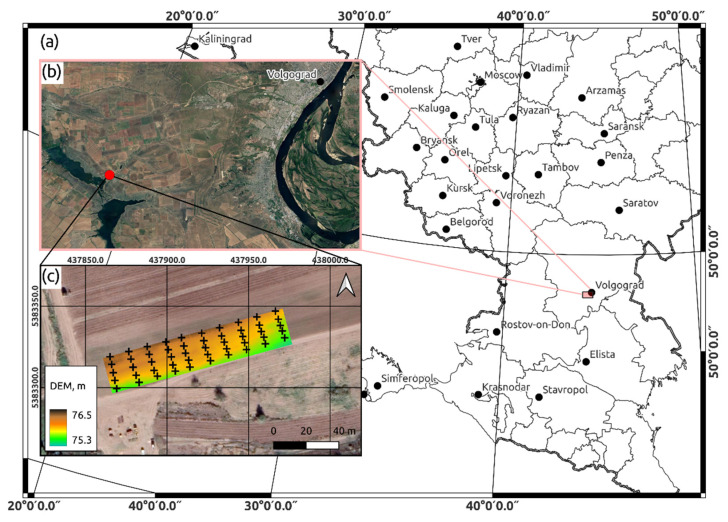
Location of the test plot: (**a**) administrative boundaries of the regions of Russia; (**b**) Google Earth imagery of the study area (red marker shows the position of Vodny settlement); (**c**) test plot with transects of soil sampling locations (+) with digital elevation model and Google Earth imagery as underlayers.

The subject of the study was the soil cover of a test plot with an area of 0.26 ha. This plot was located on the territory of the experimental production farm (EPF) “Irrigated”, owned by the All-Russian Research Institute of Irrigated Agriculture (VNIIOZ), located near the settlement of Vodny (48°35′54″ N, 44°10′17″ E), Volgogradskaya Oblast (Figure 1).

On the regional scale, the territory of the EPF “Irrigated” is located in the south of the Volga Upland in the saddle between the Volga Upland and the Ergeni Upland, which separate the drainage basins of the Volga and Don rivers. These drainage basins themselves are composed of Ergenin sands, which pass in a southerly direction into red-brown Scythian clays and loess-like loams.

The geological structure of the territory was formed in the Pliocene. During this epoch, it was completely covered by the Ergenin deposits, which were denuded at the end of the Pliocene and in the Quaternary. To date, the cover of the Ergenin deposits with a thickness of about 30–40 m has been preserved with features of the Pliocene accumulation that have not been lost. Ground waters lie deep (>10 m), as a rule, and they are weakly mineralized with a bicarbonate-sodium composition.

The climate of this territory is continental. The mean annual precipitation is around 300 mm with the average minimum and maximum temperatures of −26.1 (17 December 2016) and 40.7 °C (8 July 2020), respectively (Meteorological Website. Archive data. Available online: https://rp5.ru/ (accessed on 11 August 2022). The average annual potential evaporation values are 700–725 mm/year. It is also characterized as acutely semi-arid. During mild winter there are frequent thaws. The long summer is hot and almost without rainfall.

Experimental crops at EPF “Irrigated” are irrigated with water supplied from the Bereslavsky reservoir of the Volga-Don Canal system. The anion composition of this water is characterized by a bicarbonate-chloride-sulfate composition with SAR in the range of 3.2 ± 0.6. Its mineralization varies in the range of 0.78 ± 0.6 g/L, pH varies in the range of 8.05 ± 0.15 and the concentration of sodium ion varies in the range of 5.2 ± 0.2 mmol/L.

Four soil profiles were prepared on the territory of the EPF “Irrigated” with the aim of soil mapping. These soil profiles were located on the transect passing through the test plot area and its surroundings. According to the morphological description of these sections, it was found that the soil cover includes light chestnut-irrigated alkaline calcareous soils on yellow-brown loams (according to the soil classification of the USSR). Soils are classified according to the WRB classification as Luvic Kastanozems (Loamic, Aric, Protosodic, Bathygypsic).

The texture of the soil cover was determined using a combination of sieve and pipette (Kaczynski version) analyses. The obtained results showed that the soil particle distribution was classified as silty loam (clay loam), according to the Guidelines for the description of soils of the FAO [29].

The test site has a rectangular polygonal shape of 90 × 20 m, devoid of vegetation. Its upper border, facing northeast, adjoined soybean crops. The irrigation of this soybean crop was carried out with a frontal sprinkler machine. The mesorelief of the site is a leveled gentle slope with difference of the height 1.3 m at upper (northeast) and lower (southwest) borders, resulting in an inclination of an average of 2 degrees with a southeast exposure towards the underlying beam.

Interest in the chosen location of the test site was instigated by its periodic moistening by irrigation runoff from the upstream soybean crop. This runoff was formed during each irrigation event. Its fluxes flowed to the test site through a previously formed rill network. Part of these fluxes was transferred in a tangential direction along the transit network beyond the lower boundary. Another part was moved in a transversal direction, causing the inundation of relatively flat watershed part of test plot.

**Figure 2:**
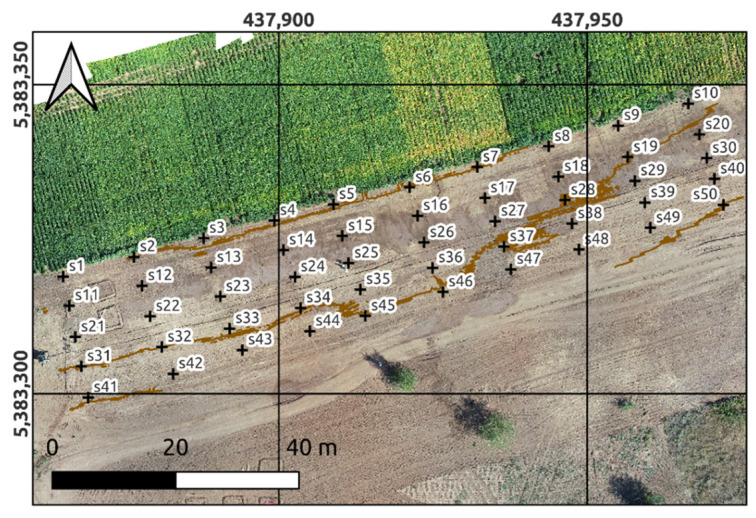
Orthophoto of the test plot with traces of surface runoff formed as a result of soybean irrigation. Brown lines represent relief isolines with contour intervals of 0.35 m.

Before the experiment, the new watering of test plot was carried out after irrigation event of neighboring crop. As a result, new irrigation runoff flows entered to the test area through several places. The contours of the humidification zones formed in this case were recorded through imaging in the visible range using a camera mounted on a UAV (Phantom 4 Pro, Dji). An orthophoto map which formed the basis of the survey results is shown in Figure 2. In this image, the watered contours are darker.

There is a high degree of probability that the identified moistening contours of the surface of the test area were caused by the wetting of deeper horizons. This was of interest when taking the measurements needed for the subsequent map modeling of soil moisture.

The basic hypothesis of this modeling was the assumption that there are links in the soil profile that contribute to the formation of moisture reserves. To identify these relationships, several mapping models have been created with a number of explanatory variables, such as:

(a) the moisture of soil samples and the coordinates of sampling places;
(b) proximal soil sensing obtained with the EM38-mk Electomagnetic Induction Meter;
(c) the characteristics of a digital elevation model (DEM) calculated from the results of stereophotogrammetric survey from a UAV;
(d) the characteristics of the relative position of the elements of the hydrological network.

### 2.2. Methods

#### 2.2.1. Input Data

Four stages of the field part of the experiment on the 21 August 2020 were implemented with aim of obtaining the input dataset of gravimetric moisture content of the soil cover at the test area, as well as datasets of explanatory variables. At its first stage, a regular rectangular grid with a cell size of 10 × 5 m was created, the coordinates of the nodes of which were determined by a high-precision RTK-GNSS antenna. At the second stage in each of the 50 nodes, soil sampling was carried out from depths of 0–15, 15–30 and 30–45 cm (Figure 2). For this, an Eijkelkamp soil auger (Eijkelkamp Agrisearch Equipment, Netherlands) was used, with the help of which soil samples were placed directly into soil weighing bottles. At the third stage, stereo photogrammetric survey was carried out using Dji Phantom IV Pro. At the fourth stage, the measurements of the electrical conductivity of the soil profile of four layers were taken at the nodes of the measurement grid. This was realized using an EM38-mk Electromagnetic Induction Meter (Geonics Ltd., Mississauga, ON, Canada).

**Figure 3:**
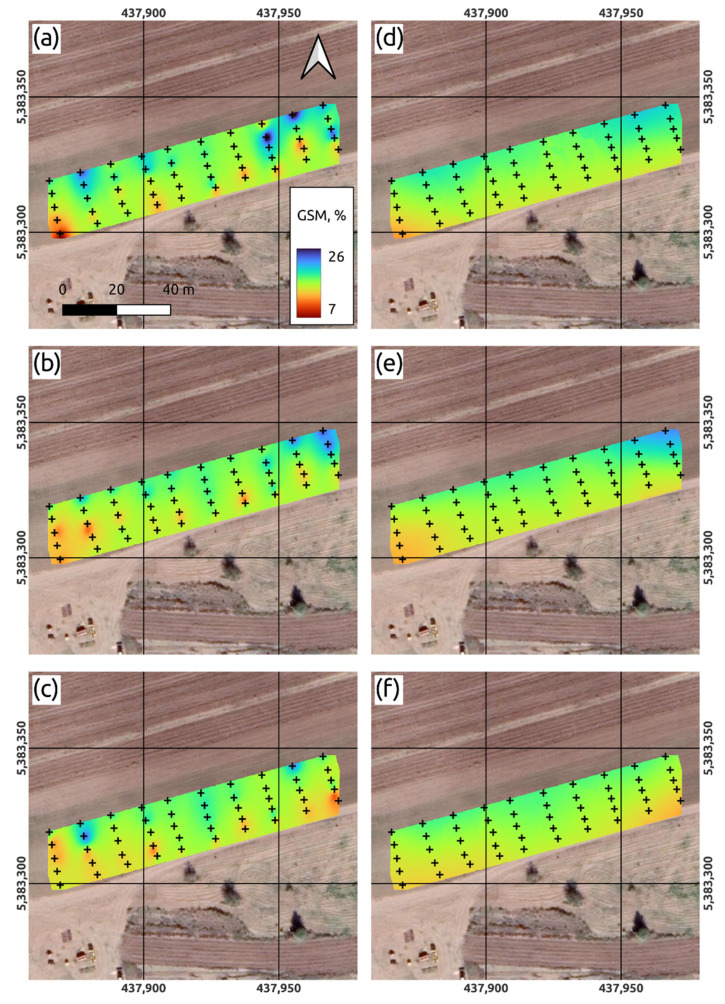
Maps of gravimetric soil moisture interpolated with inverse weighted distance (**a**–**c**) method and the deterministic Bayesian Kriging (**d**–**f**) approach on three corresponding depth intervals (0–15, 15–30 and 30–45 cm).

**Figure 4:**
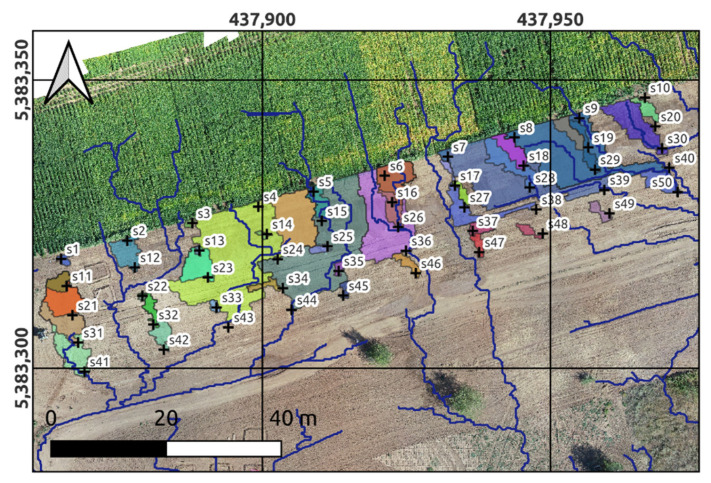
Digital hydrological model of the test plot, including the surface model, synthetic stream networks (blue lines) and its catchments within the test area (polygons).

In the VNIIOZ soil physics laboratory a standard gravimetric analysis of soil samples was applied to obtain soil moisture values for all soil samples used as the basis for following modeling. Figure 3 shows the maps of volumetric soil moisture built of each soil depth placed above orthophoto map (Figure 2). Maps at Figure 3a–c were built with the deterministic method of inverse distance weighting (IDW) and at Figure 3d–f with empirical Bayesian kriging (EBK) method. In Figure 3a–c, the minimum and maximum moisture values are noted at the control points, forming a general pattern with the presence of “hot zones”, due to the fact that IDW is a hard interpolator. The EBK maps in Figure 3d–f look smoother and do not represent the full range of moisture values. At the same time, the constructed maps, regardless of the method, weakly reflect the spatial relationship with the patterns of soil moisture reflected on the orthophoto map (Figure 2), as well as with the territories of the identified microcatchments (Figure 4).

The results of the stereo photogrammetric survey were used to obtain a digital hydrological model (DHM) of the test plot, including a digital elevation model (DEM) of the test area, as well as its spatial hydrological parameterization. The calculation of the DEM was carried out through the use of the AgiSoft PhotoScan software (Agisoft LLC, St.Petersburg, Russia) (Figure 1c) with a horizontal resolution of 2 cm, and the spatial hydrological parameterization was carried out using the Hydrological Analysis tool in ArcGIS Pro (Esri, Redlands, CA, USA). As a result, a raster layer of the elevation surface of the test plot was obtained, as well as vector layers of the 50 artificial brooks networks and boundaries of watersheds corresponding to them (Figure 4).

To conduct digital geophysical survey of the electrical conductivity of the soil cover of the test area, an EM38-mk induction reflectometer was used, comprising transmitter and receiver coils located horizontally at a distance of 1.0 m from each other. The depth of the space of soil cover affected by the measurement depends on the position of the instrument used. In the horizontal position (EC_ah_) of the EM38-mk instrument, located at a standard height of about 20 cm above the soil surface, the electrical conductivity of the 0–75 cm thickness is measured. In the vertical position (EC_av_) from the same height, this instrument measures the electrical conductivity of the 0–150 cm thickness. When measurements are carried out in both positions, from heights of more than 20 cm, the electrical conductivity of the corresponding thicknesses is measured, reduced by the difference between these heights and the standard height [30].

**Figure 5:**
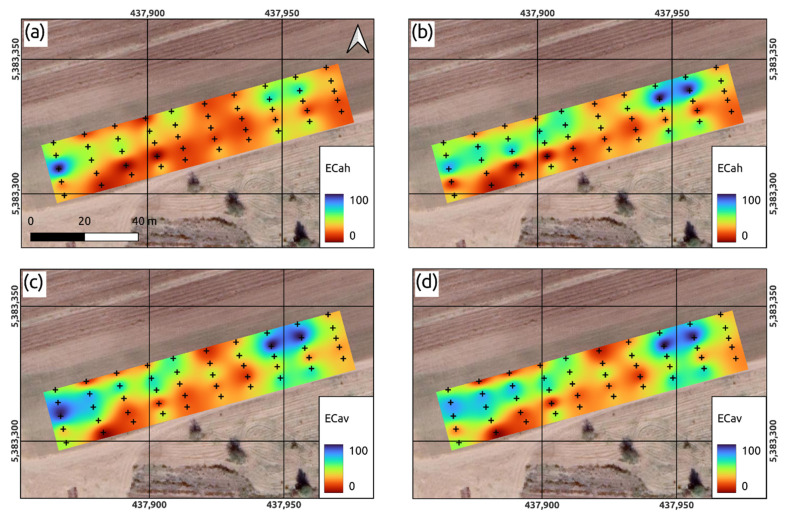
Interpolated plots of apparent electrical conductivity: (**a**) EC_ah_ measured in horizontal mode within a depth of 0–15 cm; (**b**) EC_ah_ measured in horizontal mode within a depth of 0–45 cm; (**c**) EC_av_ measured in vertical mode within a depth of 0–90 cm; (**d**) EC_av_ measured in vertical mode within a depth of 0–120 cm. Original EC values were rescaled to 0–100 for better visual representation.

Before survey, the device was calibrated at a height of 150 cm above the ground using an additional folding calibration stand. Subsequent apparent conductivity measurements were taken at all 50 soil moisture sampling sites, where all metal objects were removed to avoid interference. During the measurements, the device was elevated to the height of 30 and 60 cm from the ground in two positions—vertical and horizontal. This made it possible to obtain layers of point data of electrical conductivity corresponding to four layers of soil cover: (1) 0–15 cm; (2) 0–45 cm; (3) 0–90 cm and (4) 0–120 cm. The obtained geophysical survey results were interpolated using a spline function in SAGA GIS [31] to obtain maps of the specific permittivity (Figure 5).

The ECa maps shown in Figure 5 were obtained by the spatial interpolation of the measured point values. These raster layers were analyzed in their native spatial resolution and, if necessary, aggregated to a spatial resolution of 2 cm using the bilinear interpolation method of the GDAL library and also reprojected into the WGS84 coordinate system (UTM zone 38N, EPSG: 32638). A comparison of these four layers shows the presence of zones of similar locations with high and low EC_a_ values. At the same time, the patterns of these four layers generally have similar outlines with practically equal ranges of EC_a_. Let us assume that the corresponding values of EC_a_ depend only on the moisture content of these layers. In this case, it can be assumed that the formation of quasi-vertical zones with high values may be a consequence of a rather deep and predominantly vertical spread of infiltration flows of surface waters. The differences between these zones for different layers, both in terms of EC_a_ values and the depth of distribution, are associated with different intensities or volumes of infiltration flows in the soil cover of test plot.

#### 2.2.2. Modelling Workflow

The spatial interpolation of the gravimetric soil moisture of the test site included the following steps:

- the preparation of initial data;
- the intersection (overlay) of the existing observation points and the values of independent variables at these points (the creation of a regression matrix);
- modeling with the stepwise inclusion of independent variables using an ensemble machine learning methods and spatial cross-validation;
- the assessment of the accuracy of the obtained models;
- the spatial prediction of gravimetric soil moisture for each element (pixel) of the raster (prediction on new data).

#### 2.2.3. Ensemble Machine Learning

Ensemble machine learning (EML) methods use multiple learning algorithms to obtain better predictive performance than could be obtained from any of the constituent learning algorithms [32,33,34]. Many of the popular modern machine learning algorithms are actually ensembles. For example, Random Forest and Gradient Boosting Machine (GBM) are both ensemble learners. Both bagging (e.g., Random Forest) and boosting (e.g., GBM) are methods for assembling which take a collection of weak learners (e.g., decision tree) and form a single, strong learner. In this study, we use an ensemble machine learning algorithm called stacking (super learning, stack generalization, stack regression). The architecture of such an algorithm contains two or more base models, as well as a meta-model that combines the predictions of the base models. The steps involved in training and testing an ensemble algorithm are described below.

1. Set up the ensemble.
  (a) Specify a list of L base (null) algorithms (with a specific set of model parameters);
  (b) Specify a metalearning algorithm.
2. Train the ensemble.
  (a) Train each of the L base algorithms using the training set;
  (b) Perform k-fold cross-validation on each of these learners and collect the cross-validated predicted values from each of the L algorithms;
  (c) The N cross-validated predicted values from each of the L algorithms can be combined to form a new N × L matrix. This matrix, along with the original response vector, is called the “level-one” data. (N = number of rows in the training set);
  (d) Train the metalearning algorithm on the level-one data. The “ensemble model” consists of the L base learning models and the metalearning model, which can then be used to generate predictions on a test set.
3. Predict using new data.
  (a) To generate ensemble predictions, first generate predictions from the base learners;
  (b) Feed those predictions into the metalearner to generate the ensemble prediction.

For base or null algorithms in this study, we used Random Forest, gradient boosting machine and a generalized linear model with regularization (cvglmnet). As a metalearning algorithm, linear regression (lm) was utilized, which calculates the weighted sum of the predictions made by the base algorithms.

When using single methods based on the construction of decision trees, reliable predictions are obtained only in the area of features determined by the training sample, and in the area of extrapolation (i.e., prediction outside the training space), performance can dramatically decrease. Stack generalization, combining various models (both linear and nonlinear), decrease over-fitting and produce more realistic predictions, especially in the areas of extrapolation. Stacking takes into account the characteristics of various machine learning algorithms, reduces the variance of single models and provides better and more stable results [35]. Previously conducted studies using stacking show the potential to increase the accuracy of the produced maps of a number of soil properties [36,37].

To automate fitting of an ensemble machine learning models for the purpose of spatial interpolation/prediction, we used the “landmap” package (https://github.com/Envirometrix/landmap (accessed on 26 June 2022)) of the R statistical environment [38], which allows:

- the derivation of geographical distances (Euclidean distances to sampling sites, i.e., distances from observation sites. For each observation point, one buffer distance map is generated);
- the conversion of grids to principal components (to remove the multicollinearity effect of some independent variables);
- the automated filling of gaps in gridded data;
- the automated fitting of variogram and the determination of spatial auto-correlation structure;
- spatial overlay;
- model training using spatial cross-validation;
- model stacking, i.e., the fitting of the final EML.

#### 2.2.4. Model Evaluation

To evaluate the performance and robustness, a spatial cross-validation procedure was carried out [39]. The accuracy of the produced models was evaluated using the following metrics: the coefficient of determination (the proportion of variance explained by the meta learning algorithm) R^2^_cv_ and root mean square prediction error RMSE_cv_.

Root mean square prediction error was estimated as:

(1) $${\mathrm{RMSE}}_{\mathrm{cv}}=\sqrt{\frac{\mathrm{SSE}}{n}}$$

where

(2) $$\mathrm{SSE}=\overset{n}{\underset{i=1}{\sum }}{\left({\hat{y}}_{i}-y_{i}\right)}^{2}$$

is the sum of the squares of the cross validation error between the predicted ${\hat{y}}_{i}$ and observed values $y_{i}$ and n is total number of observations.

The coefficient of determination was estimated as:

(3) $$R_{\mathrm{cv}}^{2}=\left[1-\frac{\mathrm{SSE}}{\mathrm{SST}}\right]$$

where

(4) $$\mathrm{SST}=\overset{n}{\underset{i=1}{\sum }}{\left({\hat{y}}_{i}-{\bar{y}}_{i}\right)}^{2}$$

is the total sum of squares and ${\bar{y}}_{i}$ is the arithmetic mean of the observed values.

## 3. Results

### 3.1. Exploratory Data Analysis of Soil and Proximal Data at Sampling Sites

**Figure 6:**
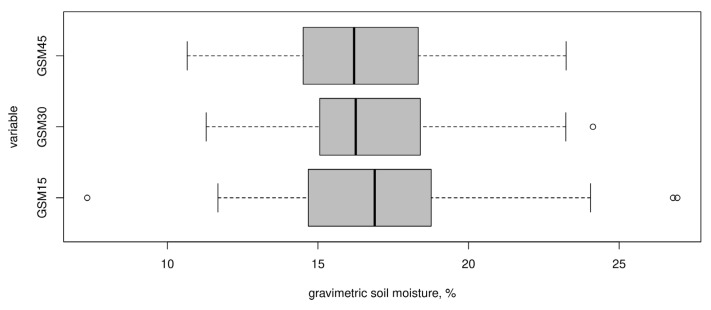
Boxplots of target variables.

Summary statistics of the gravimetric soil moisture content within the test area for a different depth is presented in Table 1 and Figure 6. The average moisture content in the upper layer (0–15 cm) was 17.09% with a minimum of 7.34% and a maximum of 26.93%. Such fluctuations in values are associated primarily with the influence of the microrelief (microridges and microdepressions between them). In sublayers 15–30 cm and 30–45 cm, the average and median values consistently decrease (16.77%, 16.25% and 16.30%, 16.20%, respectively). For these depth intervals, there is a smaller range of values between the minimum and maximum values. A non-zero positive skewness for all observed variables has been observed, which indicates a slight bias towards lower values.

The features noted above can also be seen in Figure 6, which shows the boxplot diagrams. The lower diagram clearly shows outliers that give a larger range of gravimetric moisture values in the depth interval of 0–15 cm. In general, it can be noted that the distribution of target variables is close to normal and does not require transformation.

**Figure 7:**
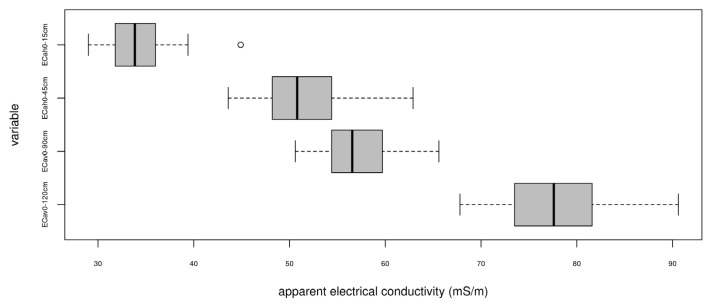
Boxplots of EM38-mk data.

Table 2 and Figure 7 summarize the statistics of electrical conductivity values measured at soil sampling sites. The highest values of electrical conductivity were obtained in the vertical mode at the sensor height above the soil surface of 30 cm (EC_av_ 0–120 cm). Thus, the average electrical conductivity was 77.59 mS/m with a minimum of 67.8 and a maximum of 90.6 mS/m. Lower electrical conductivity values were obtained in the same mode but with an instrument height above the surface of 60 cm (EC_av_ 0–90 cm): median 57.13 mS/m with a minimum of 50.6 and a maximum of 65.6 mS/m. Sufficiently close electrical conductivity values were obtained in the horizontal mode at a sensor height of 30 cm (EC_ah_ 0–45 cm): median 51.4 mS/m with a minimum of 43.6 and a maximum of 62.9 mS/m. The lowest values were obtained in horizontal mode with an instrument height of 60 cm (EC_ah_ 0–15 cm): median 33.85 mS/m with a minimum of 29.0 and a maximum of 44.9 mS/m. A possible reason for the decrease in electrical conductivity values is associated with a lower soil bulk density in the surface horizons, as a result of which the proportion of soil air increases, which has a significantly lower electrical conductivity than the solid or liquid phases of the soil. In addition, the surface horizon quickly loses moisture (due to surface runoff and evaporation), which is also an important conductor of electric current. With depth, the texture of soil changes in the direction of increasing in the proportion of clay particles with a high cation exchange capacity and, as a result, an increase in electrical conductivity is observed.

Based on the data in Table 2 and Figure 5 and Figure 7, we assume that the upper soil horizons (topsoil) were probably less conductive than the lower ones (subsurface).

### 3.2. Evaluation of Model Performance

The modeling of gravimetric soil moisture was carried out with stepwise inclusion of independent variables (covariates), using an ensemble machine learning approach and spatial cross-validation. The results of spatial cross-validation are shown in Table 3. According to the obtained results, DEM and its hydrological characteristics explain about 30% (R^2^_cv_ 0.28–0.32) of gravimetric soil moisture variability at the considered depth intervals. The further addition of geographical variables (i.e., buffer distances) to the analysis, which allows the inclusion of a spatial dependency structure, increases the prediction performance by 7–15% (R^2^_cv_ 0.39–0.43) and reduces the prediction error. The subsequent inclusion of soil electrical conductivity data into the analysis increases the prediction performance by 14–18% (R^2^_cv_ 0.53–0.57), which leads to the maximum increase in modeling accuracy with the stepwise addition of covariates. The maximum increase in the prediction performance upon including data on electrical conductivity is associated with their information content. Such data provide information not only about the surface but also allow one to “look” inside the soil thickness (within the theoretical depth) and obtain direct data on its properties. The subsequent increase in the prediction performance by 4–7% is associated with the conversion of a set of independent variables (i.e., DEM, hydrological characteristics derived from DEM, as well as data on electrical conductivity) into the principal components to eliminate the effect of multicollinearity and excessive parameterization of models. Such a transformation of covariates does not always take place, since some non-parametric methods (for example, Random Forest) have built-in protection against model overfitting. Since in this study we use the EML paradigm, which includes, among others, a linear model (which is sensitive to multicollinearity) as basic algorithms, it was decided to convert the original layers into principal components.

Produced models showed satisfactory results with the best performance of R^2^_cv_ 0.64 for the depth interval 0–15 cm, 0.59 for the depth interval 15–30 cm and 0.61 for the depth interval 30–45 cm. Combined data from the geophysical sensor (EC_av_ and EC_ah_), high-resolution topographic variables, as well as geographic variables (buffer distances) is useful for solving the problem of spatial variability in gravimetric soil moisture.

**Figure 8:**
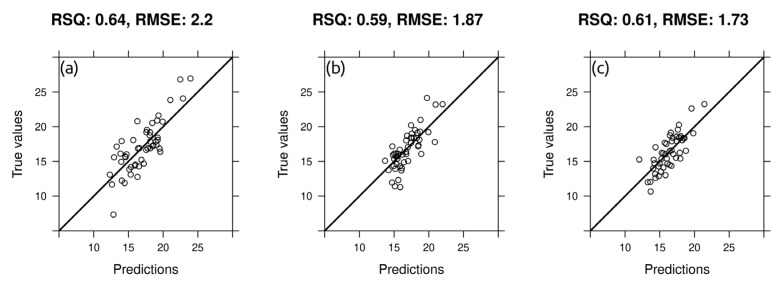
“Predicted versus measured” plots for best models based on spatial cross validation: (**a**) best model for 0–15 cm gravimetric soil moisture; (**b**) best model for 15–30 cm gravimetric soil moisture; (**c**) best model for 30–45 cm gravimetric soil moisture. Black bold line = 1:1 line. RSQ = R^2^_cv_.

The results of spatial cross-validation for the best models are shown in Figure 8. These plots reflect the correlation between the observed values versus predictions. Figure 8a shows that the low values of gravimetric soil moisture are slightly overestimated, while high values are underestimated. So, we do not observe predicted values greater than 25 and less than 10% in the depth range of 0–15 cm. However, according to Figure 6, values above 25 and 10% turn out to be outliers and are very small in the training sample. Figure 8b (depth range 15–30 cm) and 8c (depth range 30–45 cm) also show an overestimation of lower soil moisture values, which can be explained by the initial non-zero positive skewness in the distribution of moisture values. In some studies [23], it was proposed to use a logarithmic transformation of the initial data; however, we decided to use the initial values.

The RMSE_cv_ of models consistently decreases with increasing depth. Most likely, the prediction error increases as the values range of the variables increase. The best prediction results (in terms of the smallest prediction error) were obtained for the model of gravimetric soil moisture in the depth range of 30–45 cm (RMSE_cv_ ± 1.73%) where the shortest range of the values is observed (Figure 8c).

According to the results of stack generalization for the best models, it is seen that, in general, of the three base algorithms used, more weight is given to the Random Forest, followed by boosting (GBM) and a generalized linear model with regularization (cvglmnet).

## 4. Discussion

In the last two decades, there has been intensive development of the research in space soil property prediction, based on a combination of layers of geophysical data of different nature. To obtain the corresponding layers, sensors of various nature are used to measure the characteristics of linked physical processes used as predictors. As a result, systems are being created that combine sensors, including proxy sensors, as well as methods for processing data layers and using of their combinations to obtain models with higher reliability rates (R^2^_cv_, RMSE_cv_) by comparison with traditional one.

An example of such an approach can be found in the studies carried by D. Mello et al. [40]. In this work, several variants for using a number of layers of geophysical data as predictors were compared with a variant without them. The analysis of the results of these variants showed that the option without the use of proximal sensing turned out to be the least successful. Variants with additional predictors combining the data of gamma spectrometry and magnetic susceptibility of soils made it possible to obtain more reliable modeling results. This can be explained by the existence of relationships between additional data and pedogenesis and a number of the measured characteristics of the studied soils formed by it. In another study by M. Saifuzzaman et al. [41], the authors concluded that the use of soil cover electrical conductivity data, along with topographic data, provides a good opportunity to predict a number of soil characteristics needed to map their spatial variability at the field scale.

Despite the positive results of the use as predictors a spatial data layers obtained with proximal soil sensing, they are often insufficient to describe the three-dimensional variation in soil cover characteristics at required spatial scale. In this regard, in a number of studies of 3D models [42,43,44], a three-dimensional modeling of soil cover characteristics with the use of the depth of measurements as an independent variable was proposed. This approach is of particular interest in the absence of a linear relationship between the studied soil cover characteristic and the depth at which it is measured. To implement this approach, machine learning methods are used, which make it possible to create models based on nonlinear relationships in the system “soil properties–soil formation factors” at different scales.

The experimental and model results presented above correspond to a relatively small rectangular test area. This plot adjoined on its long side with a size of about 90 m to the border of the irrigated experimental soybean crop, which was located higher on the slope. The short side of the test plot with a size of about 20 m was directed to a bare, devoid of vegetation soybean sowing protective strip, limited in its lower part by a country road. As a result, the area of the formed test area turned out to be located on a part of this protective strip, which had an average slope of about 2°.

The choice of the location of the test site was directly related to the erosion network on its surface, which was formed as a result of the release of surface runoff from under the soybean crop. This runoff was a consequence of the excess of the intensity of irrigation water sprinkling of the intensity of soil infiltration. This process was led to the formation of a layer of non-infiltrated irrigation water, which sought to find ways of its runoff along the surface of the soil cover. As a result of the eight consecutive irrigations of soybean crops preceding the time of the experiment, a stable network of erosion channels was formed on the surface of the test area, through which the irrigation runoff was redistributed over its surface and also went beyond its lower and lateral boundaries.

The visual analysis of the surface of the test area indicated the presence of a number of clearly identifiable locations at the entrances of six erosion channels, through which surface runoff entered into the test area. Five of these locations were located on part of the common border of the test plot and soybean crop in the direction from the center to the east, and only one of them was located at the end of its western part. The places of its subsequent redistribution over the surface of the test area were concentrated in its middle, which were visually distinguished by a darker color. This indicated a high moisture content of the soil cover in these places in comparison with neighboring ones.

The location of darker shades in color zones (Figure 2) clearly illustrates the presence of so-called dead-end zones with stagnant water. On the whole, these zones corresponded to closed low elements of the microrelief, into which part of the surface runoff entered as it moved across the test area. The surface runoff that entered these zones was redistributed over their surface, as a result of which a layer of water was formed, which, under the influence of evaporation and filtration, penetrated deep into the soil cover.

The dead-end zones formed during the passage of surface runoff had significantly higher moisture content of the soil cover within their boundaries. The additional evidence of the formation of dead-end zones in the identified areas was a thin layer of silt particles that entered there with surface runoff and precipitated after it was infiltrated and/or evaporated.

To simulate the consequences of the runoff and the corresponding water content in the soil cover, a digital hydrological model of the erosion network of surface runoff channels and adjacent stagnant zones was created. For this purpose, a digital elevation model of the soil surface was used, which in turn was obtained from data obtained by UAV stereo survey with a planned resolution of about 1 cm. As a result of the following hydrological analysis, about 200 linear and area objects were obtained, corresponding to the system of erosion channels and dead-end zones.

Most of the derived dead-end zones were located in separate areas, which had a lighter color in comparison with the identified dead-end zones with stagnant water. This indicated that they were not involved in the moistening of the soil cover with surface runoff formed by irrigation. In this regard, these objects were excluded from the hydrological system using manual editing. The remaining objects included linear objects, which were digital models of erosion network channels, as well as areal objects, which were digital models of dead-end zones with stagnant water that had at least one connection with linear objects.

The resulting model of the hydrological network (Figure 4) is a relatively symmetrical image. Its middle part is cut by two relatively straight channels. This indicates a high velocity of water flow through these channels, which, obviously, was a consequence of the high slope of the soil cover in the respective places. On both sides of these channels, located in the center, there are two groups of channels directed in different directions and which, outside the test area, on a country road, straighten and flow into one another.

The directions of the modeled channels beyond the lower boundary of the test plot coincide with the direction of the preferred (highest) slope. Within the test plot itself, especially in its middle part, these channels have an ornate shape, which indicates the presence of the smaller slopes of the soil cover surface, and thus lower water flow rates through them. The result of this was the exit of the runoff in the transverse direction, which led to its entry into dead-end zones and their flooding with stagnant water.

In general, a visual comparison of the location and shape of the darker contours, as well as the channels of the erosion network depicted in the orthophoto (Figure 4), satisfactorily corresponds to the location of linear and area objects formed by the hydrological network tool. At the same time, it is also necessary to note an interesting feature obtained using the hydrological analysis tool used. It lies in the fact that part of the catchment area adjacent to the erosion channels located in the left and right parts of the test area, as a result of the surface runoff entering them, were flooded.

In other words, these watersheds played the role of a damper, where surface runoff rushed when high water levels in the canals were reached. In river systems, this happens during floods, when the capacity of the channel cannot cope with the incoming flow, which leads to an increase in its level and access to floodplain areas, leading to their flooding. Apparently, a similar situation took place at the level of the test plot when intense irrigation flows entered it.

The next stage of the analysis of the results of the experiment was aimed at obtaining a model of the spatial distribution of soil moisture in the soil cover of the test plot after the last case of surface runoff formation as a result of the sprinkling of soybeans adjacent to its upper boundary. The soil samples laid down for this site at 50 points of the test site were located in different places.

Some of them were in areas of dead-end zones, others in places located near the channels of the erosion network, and still others outside the boundaries of the hydrological network. This was carried out deliberately so that soil moisture data as well as electrical conductivity measured at these locations would have indirect causal relationships. The latter could be useful for displaying the influence of the formed erosion network on the measured characteristics of the soil cover.

The information usefulness of the formed spatial arrangement of sampling and measurement points was clearly confirmed as a result of the unsatisfactory spatial interpolation of gravimetric moisture values at three depths of the test area. Two interpolation tools were used to obtain soil moisture maps (Figure 3). One of them (inverse weighted distance) is based on the deterministic method and the second one (deterministic Bayesian Kriging) is based on the kriging method.

The interpolation maps obtained using the first method have a rather pronounced point orientation, especially for the upper layer of 0–15 cm, which, in general, although they satisfactorily identified places with higher and lower humidity values, did not allow obtaining a significant spatial connectedness. As a result, the obtained patterns of humidity turned out to be fragmented into small, separate interconnected areas.

In turn, the interpolation maps constructed using the second method, on the contrary, although they made it possible to focus on the formation of spatial relationships between moisture values at neighboring points, did not allow obtaining more pronounced patterns.

A slightly slower decrease in moisture content in depth in zones located near erosion channels indicates a greater moisture content of the surface layer and almost constant moisture content in depth with higher values inside dead-end zones. This indicates the potential for the deeper wetting of the soil cover in the thickness below these zones as a result of longer flooding.

A somewhat different type of map was obtained by interpolating the data of the electrical conductivity measurements of four layers of the soil cover, carried out before sampling the soils. These maps, corresponding to four soil cover layers with an upper boundary on its surface, were obtained by an interpolation tool based on a spline function (Figure 5). On the whole, the visual analysis of these maps indicates the presence of a similarity in the location of zones with high and low moisture values, which obviously resulted from an almost linear relationship between the characteristics of the soil cover.

**Figure 9:**
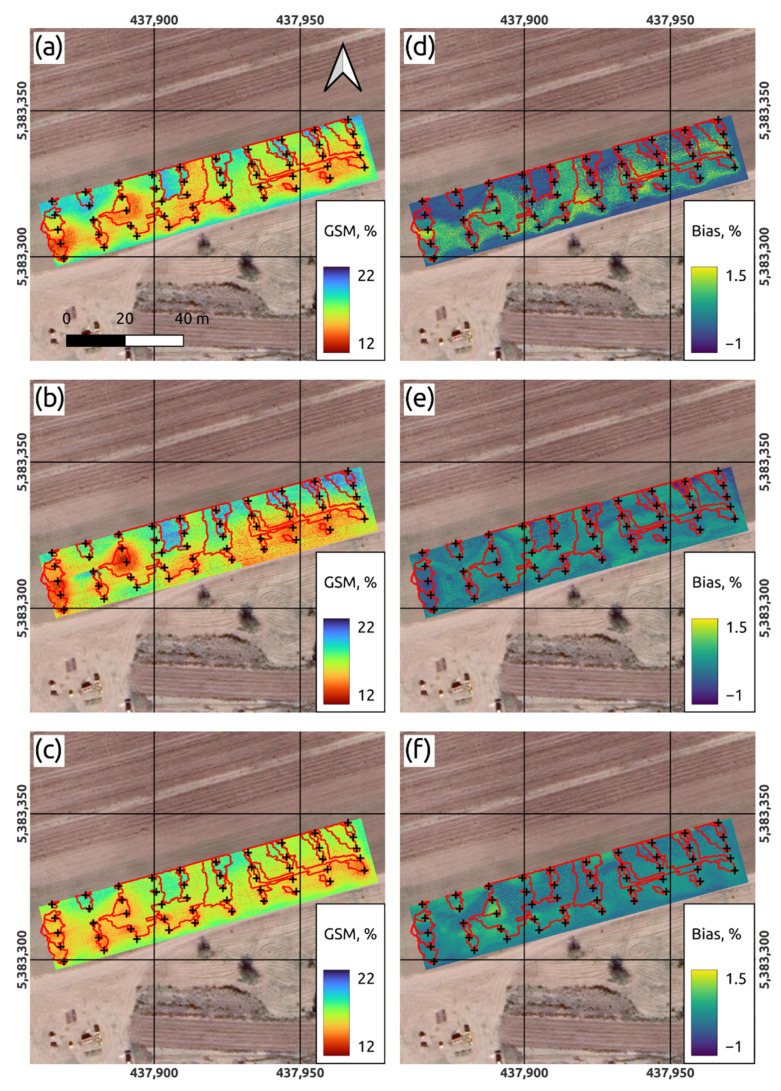
Maps of predicted gravimetric soil moisture (GSM) and their corresponding error maps: (**a**) predicted GSM at depth interval 0–15 cm; (**b**) predicted GSM at depth interval 15–30 cm; (**c**) predicted GSM at depth interval 30–45 cm; (**d**) conditional bias at depth interval 0–15 cm; (**e**) conditional bias at depth interval 15–30 cm; (**f**) prediction conditional bias at depth interval 30–45 cm.

Figure 9 shows maps of gravimetric soil moisture over a number of depth intervals. The map in Figure 9a shows the gravimetric moisture content of the soil in the depth range of 0–15 cm. On the corresponding map, zones of high and low values are clearly distinguished, as well as a tendency to decrease in moisture values with distance from the border of the field with irrigated soybeans. The zones of high humidity values are confined to microcatchments that do not have surface runoff through a network or with runoff through weakly expressed microrills. Here, high moisture values can be associated with the outfall of the stream network from under the soybean crop, the surface runoff of which is either a result of the formation of irrigation runoff or of intense rainfall. Partly, this may also be explained by the partial moistening of the border part of the test area during soybean irrigation and the absorption of part of the water entering the test area as a result of surface runoff. The map also clearly shows a pattern of trickle rills (extensional and transverse), which are of anthropogenic genesis and are characterized by higher humidity values than the surrounding microhills. It is also possible to observe the absence of predicted moisture values below 10 and above 25% on the map. However, according to the boxplot (Figure 6, lower plot), the range of predicted values (from 12 to 22%) fits well into the range. The average predicted value of gravimetric soil moisture according to the obtained map is 16.71%, which is very slightly different from the true average value at this depth interval—17.09% (Table 1).

The maps in Figure 9b,c show the gravimetric soil moisture in depth intervals of 15–30 cm and 30–45 cm, respectively. In general, the pattern of higher and lower soil moisture values in the considered depth intervals is similar to the pattern in the 0–15 cm interval, which indicates the similarity of moisture values and, probably, the virtual absence of lateral soil runoff. Figure 9b shows some “hot spots” with the lowest humidity values, which is apparently due to the large influence of geographical variables (buffer distances). A regular slight decrease in soil moisture values with increasing depth can be noted. The average predicted values of gravimetric soil moisture according to the produced maps are 16.48 and 16.45% in the depth intervals of 15–30 cm and 30–45 cm, respectively.

The maps in Figure 9d–f show the bias, i.e., the mathematical expectation of the difference between predicted and true values. We can note a negative bias for areas of high values of moisture in the depth interval of 0–15 cm, which confirms the conclusion that they are underestimated (with maximum of 1%), and also note a positive bias for areas of low values, which indicates their overestimation (with maximum at 1.5%). We can also observe a lower bias of the predicted values from the true values of gravimetric moisture in the intervals of 15–30 and 30–45 cm.

The accuracy of the models turns out to be satisfactory, which is due to the use of the spatial cross-validation approach. In the case of randomly splitting, spatial data can lead to training data, which are neighbors in space with test data. Due to spatial autocorrelation, test and training datasets would not be independent in this scenario, with the consequence that cross-validation fails to detect a possible overfitting. Spatial cross-validation alleviates this problem.

As mentioned above, the experimental and model results presented correspond to a relatively small test area. When transferring the models outside the training locations (i.e., into a new geographic space), it is assumed that the learned relationships between covariates and responses still preserved. However, especially in heterogeneous conditions, the new geographical space may differ significantly in its soil properties from what was observed in the training data. Therefore, gaps in the covariate space where there is no support of training data must be considered problematic because the algorithm was not enabled to learn about the relationships in these environment. In this regard, a delineation of the area to which a prediction model can reliably be applied is required (“area of applicability”) [45]. Predictions that go beyond the area of applicability should be handled carefully or excluded from further consideration, since the properties of environment are too different from those observed in the training data. It is obvious that the models we have obtained can be transferred to territories where the properties of the covariates will not differ dramatically from the training data, i.e., in territories with similar conditions in climate, relief and soil properties. In case of a significant difference in the above conditions, additional research is necessary.

Our initial predictions probably will not be correct enough to immediately support informed farm-scale irrigated management. However, we can offer our initial predictions as a starting point.

## 5. Conclusions

This is the first study focused on the possible use of a machine learning approach for the purpose of connecting data of the soil cover moisture with topographic derivatives and electrical conductivity data on agricultural land in the Volgograd region. The obtained cartographic models showed satisfactory results with the best performance R^2^_cv_ 0.59–0.64. The RMSEcv of models consistently decreases with increasing depth. It is probable that the prediction error increases as the values range of the variables increase. The best prediction results (in terms of the smallest prediction error) were obtained for the model of gravimetric soil moisture in the depth range of 30–45 cm (RMSEcv ± 1.73%), where the shortest range of the values is observed.

The main feature of the developed approach was the use some geophysical predictors for mapping the moisture of soil cover over an area of the test plot.

Despite the relatively small spatial extent of the test plot, its surface layer had zones with different soil moisture values, which were very well reproduced using the proposed method based on machine learning of two additional datasets.

The data of contactless proximal probing play an important role in the prediction of soil properties. The results obtained indicate a significant potential for the use of PSS technologies for assessing (predicting) soil moisture in the conditions of the dry steppe of the Volgograd region. Combining the data measured by a geophysical sensor with topographic and geographical (hydrological) variables made it possible to obtain more reliable results in assessing the spatial variability of soil moisture under the conditions of surface runoff formation.

This ongoing research work will allow us to explore the possibilities of using remote sensing methods, together with the existing groundwork. The cartographic models of soil moisture variability obtained in this way can be used to solve the problem of precise irrigation and fertilization with a variable rate, thereby optimizing the use of resources by crop producers, which ultimately can lead to the sustainable management of natural resources.

## Figures and Tables

**Table 1:** Summary statistics of the gravimetric soil moisture (%) content of the test area for different depth intervals.

	0–15 cm	15–30 cm	30–45 cm
n	50	50	50
mean	17.09	16.77	16.30
sd	3.70	2.81	2.61
median	16.88	16.25	16.20
min	7.34	11.29	10.66
max	26.93	24.13	23.24
range	19.59	12.84	12.58
skewness	0.44	0.41	0.27
kurtosis	0.87	0.22	−0.02
se	0.52	0.40	0.37

**Table 2:** Summary statistics of electrical conductivity values (EC_a_, mS/m), collected with EM38-mk at sampling sites.

	EC_av_ 0–120 cm ^1^	EC_av_ 0–90 cm	EC_ah_ 0–45 cm	EC_ah_ 0–15 cm
n	50	50	50	50
mean	77.59	57.13	51.40	33.96
sd	5.47	3.66	4.37	2.84
median	77.6	56.55	50.80	33.85
min	67.8	50.60	43.60	29.00
max	90.6	65.60	62.90	44.90
range	22.8	15.00	19.30	15.90
skewness	0.29	0.49	0.47	1.09
kurtosis	−0.62	−0.46	−0.04	2.59
se	0.77	0.51	0.61	0.40

^1^ limits of theoretical depth.

**Table 3:** Spatial cross validation results of manual stepwise including the independent variables.

Target Variables	Independent Variables	R^2^_cv_	RMSE_cv_
GSM 0–15	DEM and derivatives	0.28	3.29
	+buffer distances	0.43	2.62
	+EC_a_ data	0.57	2.50
	PC ^1^ + buffer distances	0.64	2.24
GSM 15–30	DEM and derivatives	0.31	3.13
	+buffer distances	0.39	2.78
	+EC_a_ data	0.53	2.19
	PC + buffer distances	0.59	1.87
GSM 30–45	DEM and derivatives	0.32	3.31
	+buffer distances	0.39	2.74
	+EC_a_ data	0.57	2.01
	PC + buffer distances	0.61	1.73

^1^ Independent variables grids converted to principal components.

## Data Availability

The R code and datasets generated during and/or analyzed during the current study are available from the corresponding author on reasonable request. Source data are contained within the article.
